# Decision-Making in the Surgical Management of Rigid Congenital Spinal Deformities: The Role of Vertebral Column Resection and Less Invasive Alternatives

**DOI:** 10.3390/jcm15124633

**Published:** 2026-06-15

**Authors:** Piotr Kowalski, Justyna Walczak, Krzysztof Zakrzewski, Paweł Grabala

**Affiliations:** 1 Department of Neurosurgery, Regional Specialized Hospital, ul. Dekerta 1, 66-400 Gorzow, Poland; pkowal72@gmail.com; 2 Department of Neurosurgery, Polish-Mother’s Memorial Hospital Research Institute, Rzgowska 281/289, 93-338 Lodz, Poland; justyna.walczak@iczmp.edu.pl (J.W.); krzysztof.zakrzewski@iczmp.edu.pl (K.Z.)

**Keywords:** rigid spinal deformity, vertebral column resection, posterior vertebral column resection, halo-gravity traction, temporary internal distraction rods, asymmetric pedicle subtraction osteotomy, congenital kyphoscoliosis

## Abstract

**Background:** Vertebral column resection (VCR) has historically been recognized as the most efficacious corrective intervention for severe rigid spinal deformities. Nevertheless, advancements in preoperative optimization, staged corrective methodologies, osteotomies, and contemporary instrumentation have broadened the spectrum of therapeutic options available. The definitive role of VCR in the modern management of rigid congenital spinal deformities remains a topic of ongoing scholarly discourse. **Methods:** This study presents two illustrative cases of severe congenital spinal deformities that were addressed employing various surgical methodologies, alongside a comprehensive review of the current literature pertaining to VCR and less invasive alternatives, including halo-gravity traction (HGT), temporary internal distraction techniques, pedicle subtraction osteotomy (PSO), asymmetric pedicle subtraction osteotomy (APSO), and multi-rod constructs. **Results:** The cases elucidated herein underscore the necessity for treatment strategies to be tailored specifically to the characteristics of the deformity, its flexibility, the neurological risks involved, and the individual patient’s specific attributes. In one case, significant deformity correction achieved via preoperative HGT facilitated successful management through multilevel Ponte osteotomies and posterior spinal fusion, thereby obviating the need for VCR. In other patient suffering from severe rigid congenital kyphotic deformity with pronounced anterior column deficiencies, VCR was deemed essential to realize adequate correction and neural decompression. All patients exhibited substantial radiographic correction, enhancements in health-related quality-of-life metrics, diminished disability and pain, while maintaining correction without neurological complications or implant failure at the final follow-up evaluation. **Conclusions:** VCR continues to be a vital element within the surgical repertoire for the treatment of severe rigid spinal deformities; however, it should not be deemed obligatory in every instance. Diligent preoperative evaluation, staged correction methodologies, and less invasive osteotomy techniques may permit satisfactory correction while mitigating surgical morbidity in suitably selected patients. Treatment approaches should be customized, favoring the least invasive procedure capable of achieving safe and lasting correction whenever practicable.

## 1. Introduction

Severe rigid spinal deformities represent some of the most challenging conditions encountered in contemporary spine surgery. These deformities, whether congenital, developmental, or post-traumatic in origin, are typically characterized by a coronal and/or sagittal Cobb angle exceeding 90–100° and less than 25% flexibility on bending or traction radiographs. Their marked rigidity, multiplanar nature, and frequently altered vertebral anatomy often limit the effectiveness of conventional corrective techniques and increase the risk of neurological complications during surgical treatment [1,2,3,4,5,6,7,8,9,10]. For decades, Vertebral Column Resection (VCR) and its posterior-only modification (PVCR) have been regarded as the most powerful corrective procedures for severe rigid spinal deformities. By allowing complete three-column resection at the apex of the deformity, VCR enables substantial correction in both the coronal and sagittal planes, with reported correction rates ranging from 54% to 67.2% and from 60% to 90.9%, respectively [9,10,11,12,13,14]. However, these benefits are accompanied by considerable surgical morbidity, including prolonged operative times, substantial blood loss, and a high incidence of neurological complications up to 69.2% [11,12,14,15,16,17,18,19,20,21,22,23,24,25,26]. Growing recognition of the risks associated with VCR has stimulated the development of alternative treatment strategies aimed at achieving satisfactory deformity correction while reducing operative morbidity. These include preoperative halo-gravity traction, temporary internal distraction rods, magnetically controlled growing rods used for staged correction, incremental osteotomies, and less extensive vertebral resections. Advances in imaging, neuromonitoring, perioperative care, and staged correction protocols have further expanded the range of available treatment options, allowing surgical strategies to be tailored to individual patient characteristics and risk profiles [27,28,29,30,31,32,33,34,35,36,37,38].

The primary aim of this study, combining the presentation of two surgically treated cases of congenital scoliosis with a review of the contemporary literature, is to evaluate whether routine application of VCR is justified for all severe rigid spinal deformities or whether alternative strategies can provide comparable deformity correction with lower morbidity and neurological risk while maintaining satisfactory clinical and radiographic outcomes.

## 2. Methods

This narrative review was conducted to examine contemporary surgical strategies for congenital and rigid spinal deformities in pediatric patients. The review specifically concentrated on congenital scoliosis and various rigid spinal deformities necessitating surgical intervention, with particular emphasis on operative strategies, indications, outcomes, and complications. A non-systematic thematic literature search was performed using PubMed/MEDLINE and Google Scholar. The search strategy was designed to identify studies involving human subjects, encompassing both pediatric and adult populations. Relevant publications were retrieved through the application of diverse keyword combinations pertaining to congenital spinal deformities, congenital scoliosis, rigid scoliosis, spinal osteotomies, vertebral column resection, hemivertebra excision, growth-friendly surgical techniques, spinal fusion, deformity correction, and surgical outcomes. To encompass the full spectrum of currently accessible surgical methodologies, the literature was scrutinized across several pivotal domains, which included surgical indications, preoperative planning, operative techniques, radiographic and clinical outcomes, as well as procedure-related complications. Additional pertinent studies were pinpointed through citation tracking and manual examination of references from significant review articles and landmark publications within the field. Study selection was executed subsequent to an initial filtration of titles and abstracts, followed by a comprehensive full-text evaluation of potentially relevant articles. Publications were deemed eligible for inclusion if they provided substantive information regarding the surgical treatment of congenital or rigid spinal deformities while reporting on technical specifications, clinical outcomes, or complications. Both original research articles and high-quality review papers were taken into consideration. Studies published between January 2000 and March 2026 were considered. Special emphasis was placed on significant complications, which included neurological injury, implant-related failure, infection, loss of correction, and the necessity for revision surgery. As this review was conducted in a narrative format, no formal methodological quality assessment or risk-of-bias evaluation was undertaken. The narrative approach was deliberately selected to enable a broad and clinically relevant synthesis of the extant evidence, facilitating the integration of historical advancements, contemporary surgical paradigms, and emerging therapeutic strategies. Rather than delivering a comprehensive systematic analysis of the literature, this review aspires to summarize and critically contextualize the prevailing knowledge regarding the surgical management of congenital and rigid spinal deformities across diverse age cohorts. For the purposes of this review, severe rigid spinal deformity refers to a deformity exceeding 90° in the coronal and/or sagittal plane with less than 25% flexibility on dynamic radiographs consistent with definitions commonly used in the spinal deformity literature. For the purposes of this publication, data collection and calculations were performed using Microsoft Excel (MS Office 2026), and Microsoft PowerPoint and Microsoft Word were used in the preparation of the manuscript.

## 3. Case Presentations

### 3.1. Case I: A 15-Year-Old Male with Progressive Congenital Kyphoscoliosis Treated Without Vertebral Resection

**Figure 1:**
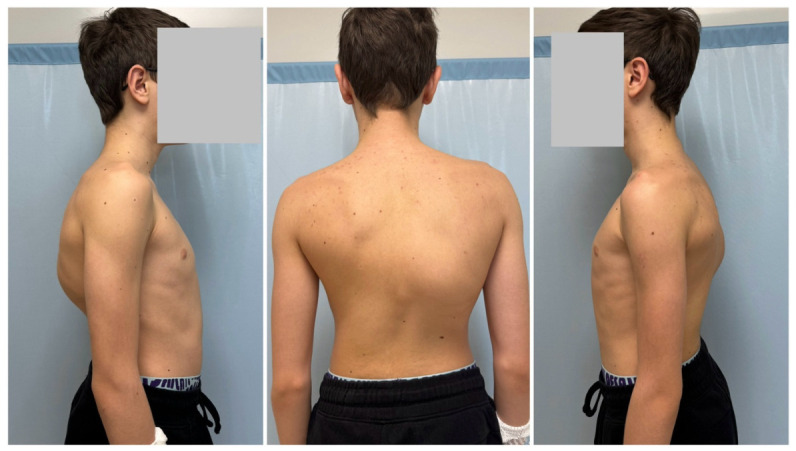

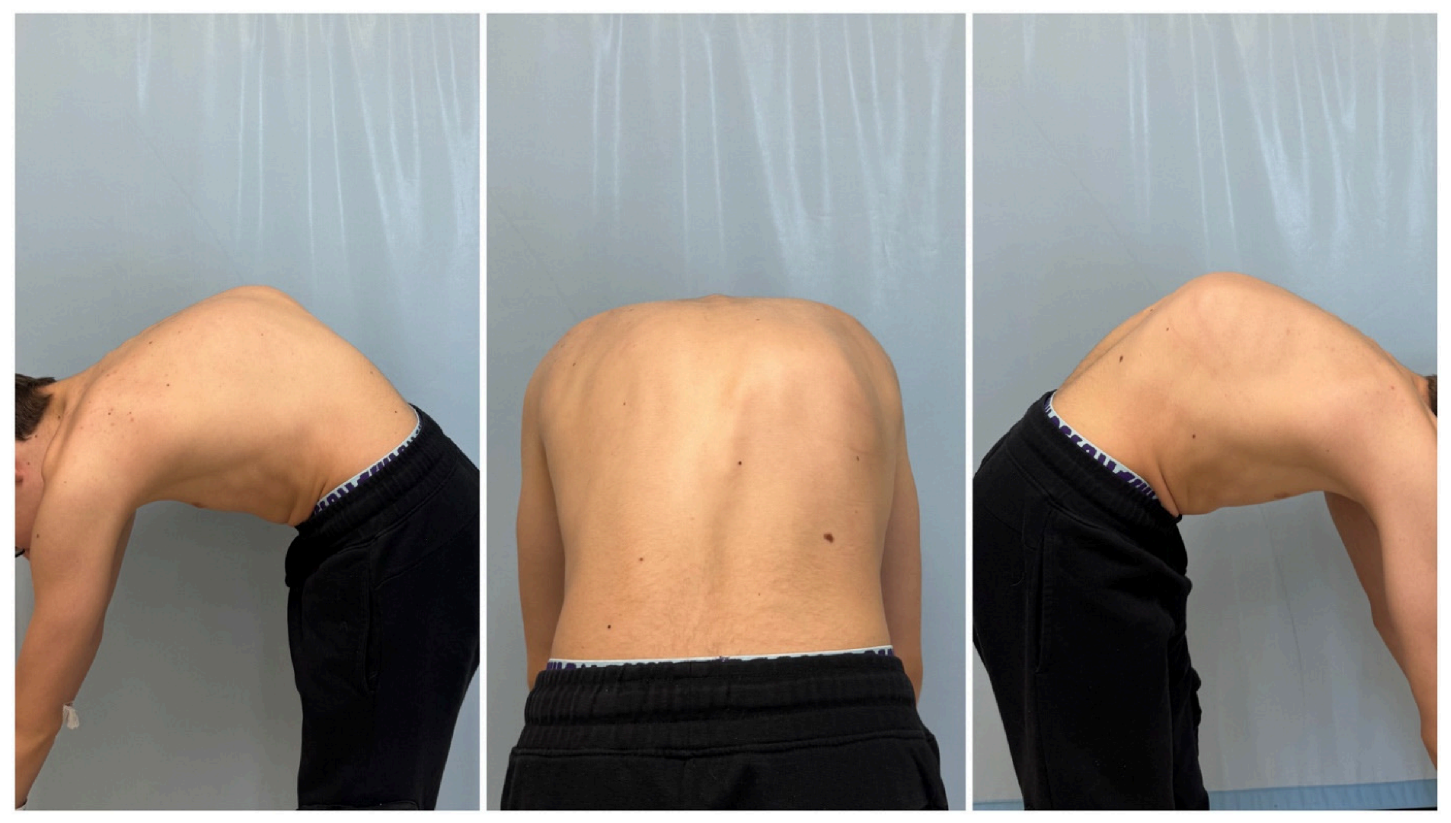
Clinical pictures of a 15-year-old boy with a progressive congenital spinal deformity that had first been detected about three months before consultation.

A 15-year-old boy presented to our pediatric orthopedic outpatient clinic, along with his parents, for an assessment of a progressive spinal deformity that had first been detected about three months prior to this consultation. On the first clinical assessment, physical examination showed a noticeable spinal deformity with typical features typical of kyphoscoliosis (Figure 1).

**Figure 2:**
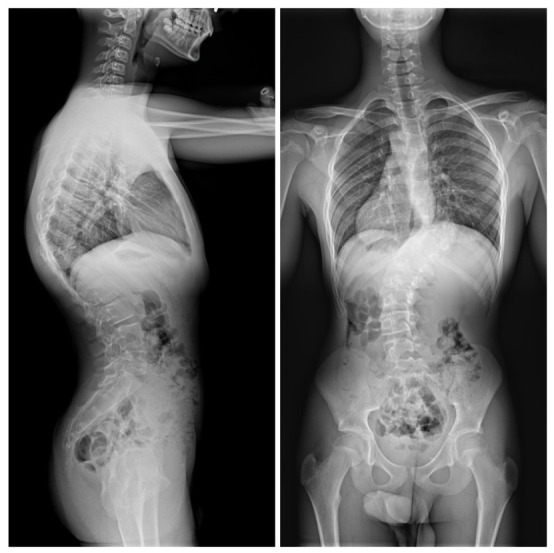
Standing posteroanterior and lateral full-spine radiography showed a congenital vertebral anomaly, consisting of a mid-thoracic (T9–T10 level) hemivertebra, leading to a progressive structural kyphoscoliotic deformity.

The deformity did not appear painful, and the patient showed a fully unremarkable neurological examination, consisting of intact motor strength, nominal sensory performance, and symmetrically brisk deep tendon reflexes across all extremities. Forward bend testing showed a notable thoracic gibbus, and clinical photography illustrated marked truncal asymmetry. The patient had full physical mobility without limitations, with no reported activity of daily living limitations and without evidence of restrictive pulmonary disease. No other diseases had been diagnosed. Standing posteroanterior and lateral full-spine radiography showed a congenital vertebral anomaly, consisting of a mid-thoracic (T9–T10 level) hemivertebra, leading on to a progressive structural kyphoscoliotic deformity. Thoracic curve magnitude was 80° using the Cobb method, with accompanying thoracic kyphosis of 72° (normal range: 20–40°) as shown in Figure 2. Supine bending films showed restricted flexibility, with correction of the curve on maximum effort for bending being just 68°.

**Figure 3:**
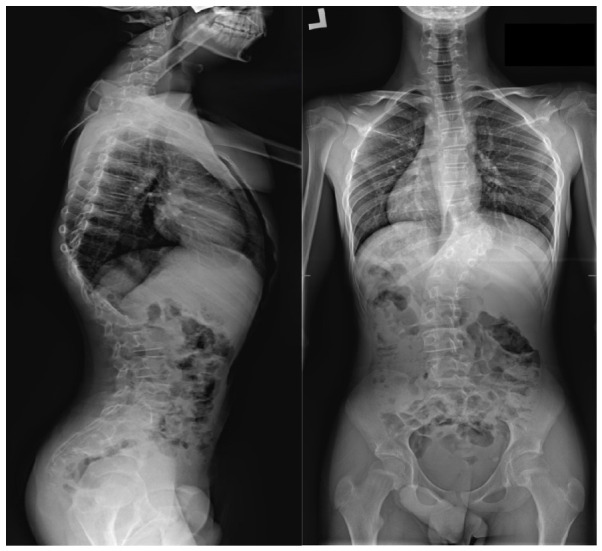
Standing posteroanterior and lateral full-spine radiographs obtained after 6 weeks of HGT demonstrated substantial correction of the deformity. At the end of the traction phase, the thoracic curve improved from 80° to 59°, representing a 27% correction. Thoracic kyphosis decreased from 72° to 42°, indicating marked improvement of the sagittal deformity.

Magnetic Resonance Imaging (MRI) effectively ruled out the presence of intraspinal abnormalities, whereas Computed Tomography (CT) substantiated the existence of a congenital hemivertebra at the T9–T10 level characterized by a triangular crossover morphology and asymmetric growth plates [39]. Subsequent to comprehensive discussions with the patient and their family concerning the natural progression of congenital kyphoscoliosis and the range of therapeutic options available, surgical intervention preceded by halo-gravity traction (HGT) was advocated. HGT was chosen due to the pronounced rigidity exhibited on bending radiographs, the patient’s youthful age and residual growth potential, as well as the objective of mitigating the complexity and neurological risks associated with definitive correction. Even though posterior spinal fusion was technically viable, it was anticipated to primarily yield in situ stabilization with minimal correction of deformity. Consequently, a deformity release along with partial vertebral resection was deemed essential to attain an acceptable alignment. Preoperative assessments of pulmonary function indicated no clinically significant restrictive impairment, thus negating the necessity for specialized respiratory optimization. The patient underwent a standard anesthetic evaluation accompanied by routine perioperative respiratory management [39,40,41]. The patient successfully endured six weeks of HGT without experiencing any major complications. At the conclusion of traction, the thoracic curvature exhibited an improvement from 80° to 59° (27% correction), while thoracic kyphosis diminished from 72° to 42° (Figure 3). Notable clinical enhancements in truncal balance and a reduction in the thoracic gibbus were also documented [42,43,44,45].

**Figure 4:**
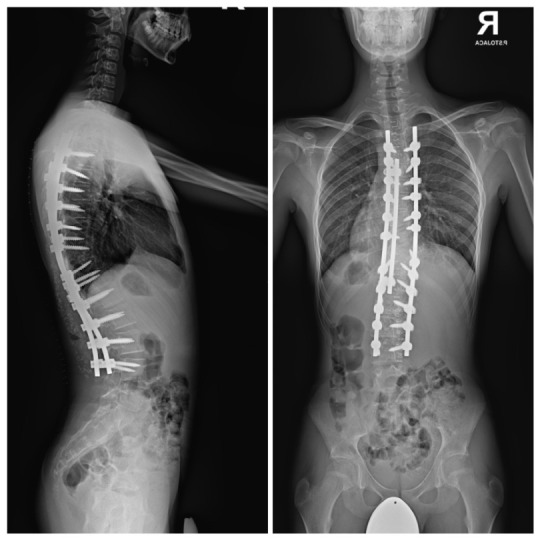
Standing posteroanterior and lateral full-spine radiographs obtained just after surgical correction (first standing X-rays). Thoracic kyphosis decreased from 72° to 42°, and main thoracic curve decreased from 80° to 22°, indicating marked improvement of the sagittal and coronal deformity.

Given the significant enhancement in curve flexibility and the reduction in deformity attained through HGT, the initially planned vertebral column resection was retracted in favor of a less invasive approach comprising multilevel Ponte osteotomies and posterior spinal fusion. This determination was predicated on the enhanced flexibility, diminished curve magnitude, and the imperative to mitigate surgical morbidity while ensuring adequate correction. Fusion was executed from T4 to L4. Pedicle screw fixation was achieved using the Reline^®^ spinal system (Globus Medical, Inc., Audubon, PA, USA).The upper instrumented vertebra (UIV) was designated as T4 to facilitate stable fixation above the structural curve and to avert concluding the construct within a kyphotic transition zone, whereas the lower instrumented vertebra (LIV) was selected as L4 to sustain coronal and sagittal equilibrium and to attenuate the risk of distal junctional failure. The procedure encompassed six Ponte osteotomies (T6–T11), segmental pedicle screw instrumentation, posterior fusion utilizing both autograft and allograft bone, along with continuous intraoperative neuromonitoring employing somatosensory-evoked potentials (SSEPs) and motor-evoked potentials (MEPs). The surgical intervention was concluded without any complications. The operative duration was documented at 6.5 h, with an estimated blood loss of 800 mL, and neuromonitoring exhibited stability throughout the entirety of the procedure. Postoperative radiographs indicated a restoration of both coronal and sagittal alignment (Figure 4). The thoracic curve exhibited an improvement from 80° to 22° (67% correction relative to the pre-traction measurement), whereas thoracic kyphosis showed an enhancement from 72° to 42°, thereby reinstating physiological sagittal alignment.

**Figure 5:**
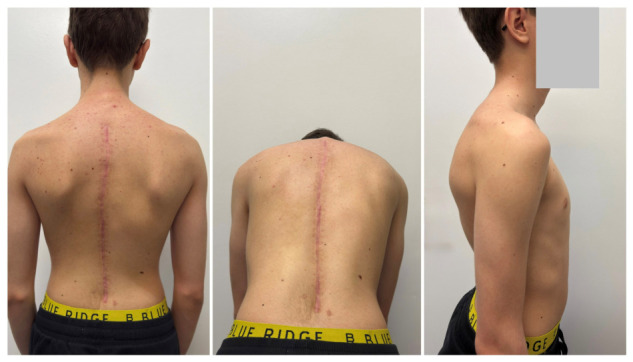
Clinical pictures obtained at six-month post-operatively showed well-balanced spine and full unrestricted physical activity which included competitive sport activity.

The patient had an otherwise uncomplicated postoperative course and was discharged home on the fifth day post-operatively with proper activity restrictions and follow-up instructions. At three-month follow-up, the patient had returned to full function, participating in daily activities which included schooling as well as active hobbies. At six-month post-operatively, he had come back to full unrestricted physical activity which included competitive sport activity (as shown on Figure 5).

**Figure 6:**
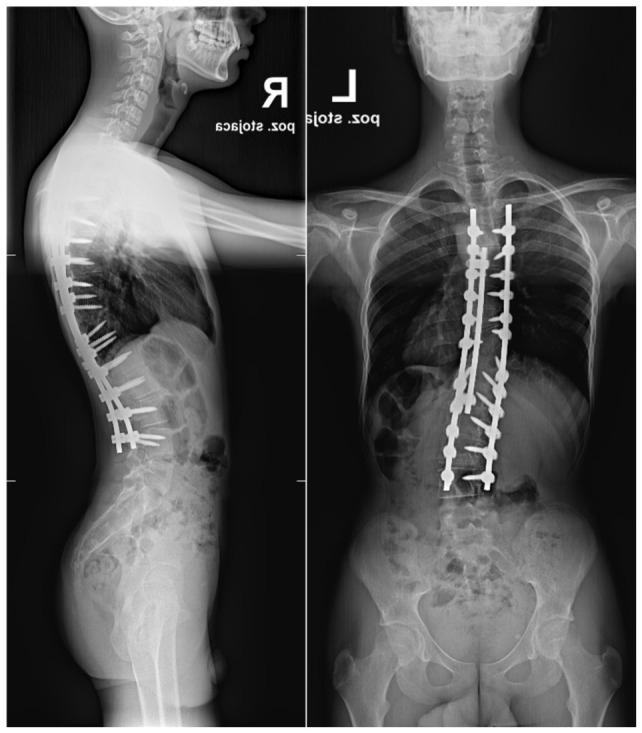
Standing posteroanterior and lateral full-spine radiographs obtained at a 2-year of follow-up.

**Figure 7:**
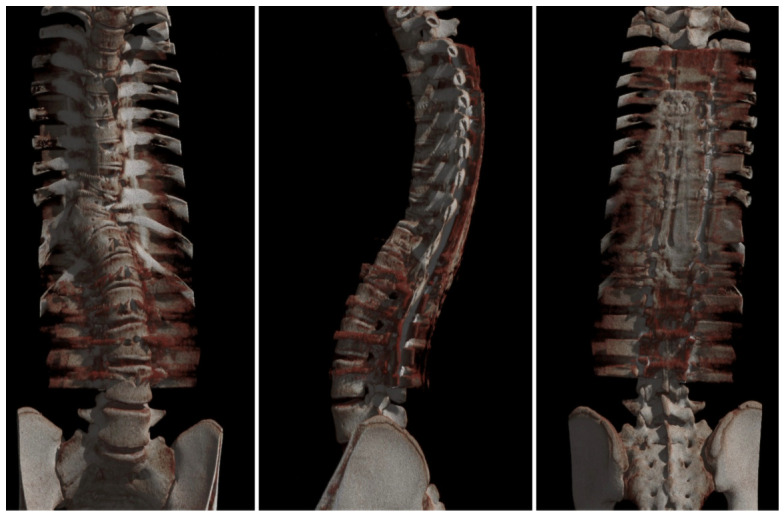
The imaging an CT obtained at a 2-year of follow-up showed posterior spinal fusion.

The patient, as well as his family, both showed extremely high level of satisfaction with both the functional and cosmetic results of the surgery. Serial follow-ups in the form of radiograph imaging showed maintenance of correction without demonstration of what could be called implant-related complications or loss of alignment. Fusion at site was shown by X-ray imaging an CT on follow-through at a 2-year follow-up (as presented in Figure 6 and Figure 7), as well as a balanced spine.

### 3.2. Case II: A 13-Year-Old Female with Progressive Congenital Kyphosis Treated with Vertebral Resection and Anterior Column Reconstruction

**Figure 8:**
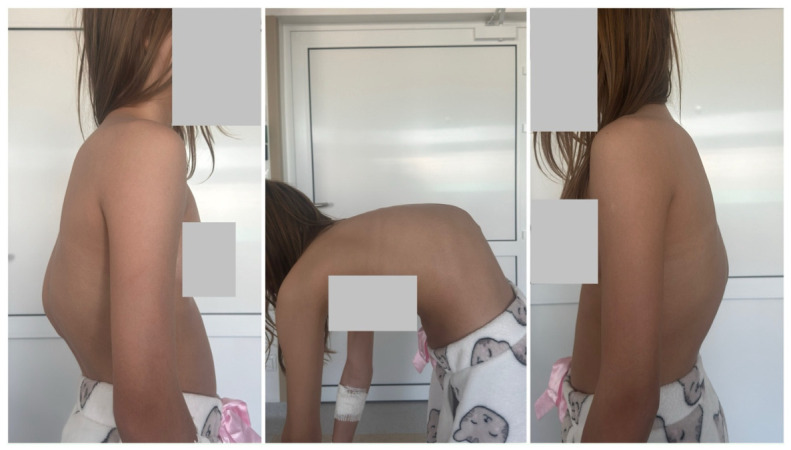

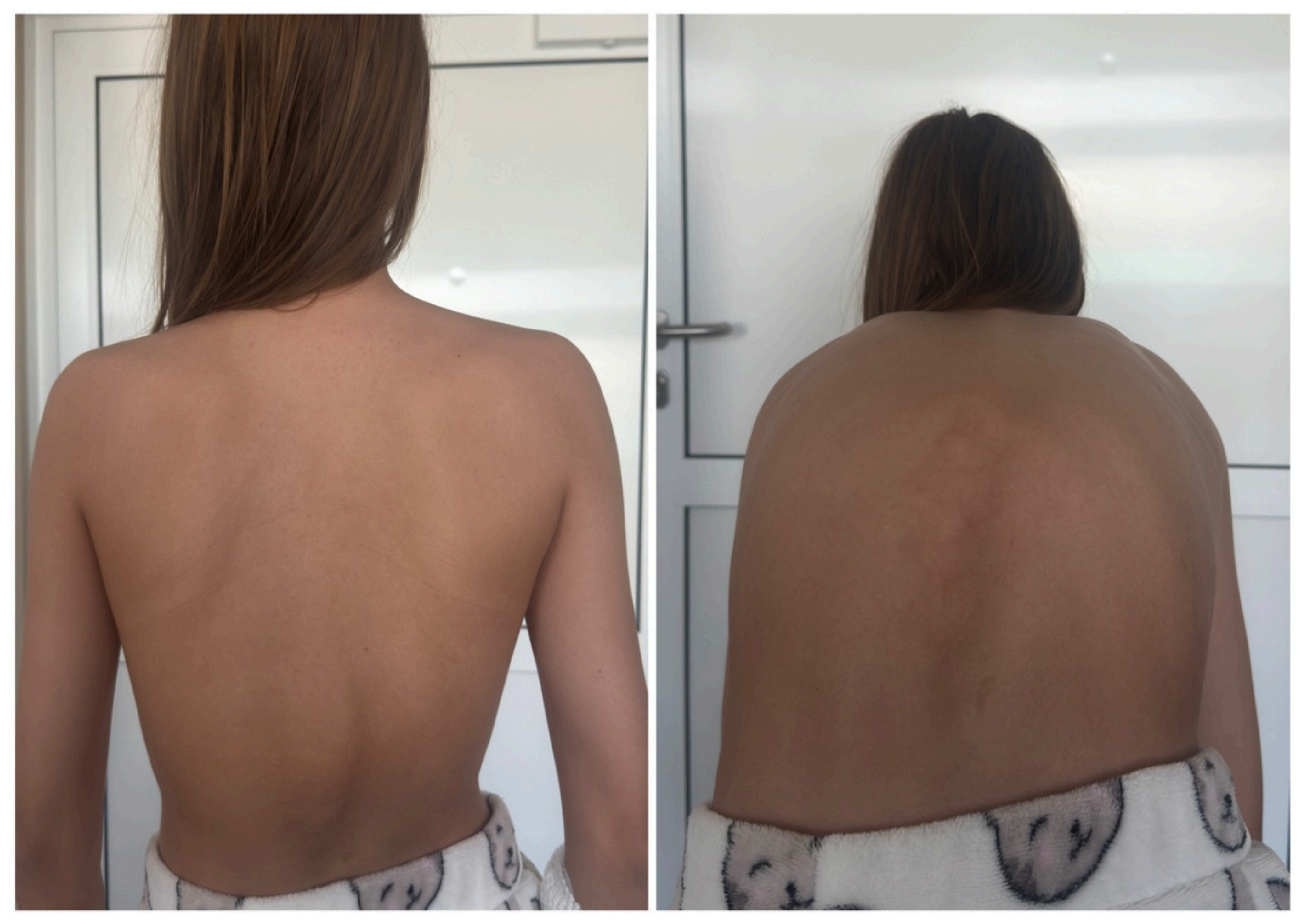
Clinical pictures of a 13-year-old girl with a progressive congenital spinal deformity.

A 13-year-old female patient was referred to our pediatric spine clinic due to a progressive congenital thoracolumbar deformity that exhibited an increase from 65° to 92° over a two-year duration, despite consistent orthopedic monitoring. Although she did not report experiencing back pain, neurological symptoms, or any restrictions in her daily activities, she had manifested mild exercise intolerance and exertional dyspnea. A clinical examination revealed a notable angular gibbus and truncal imbalance (Figure 8). The neurological assessment yielded normal results. 

**Figure 9:**
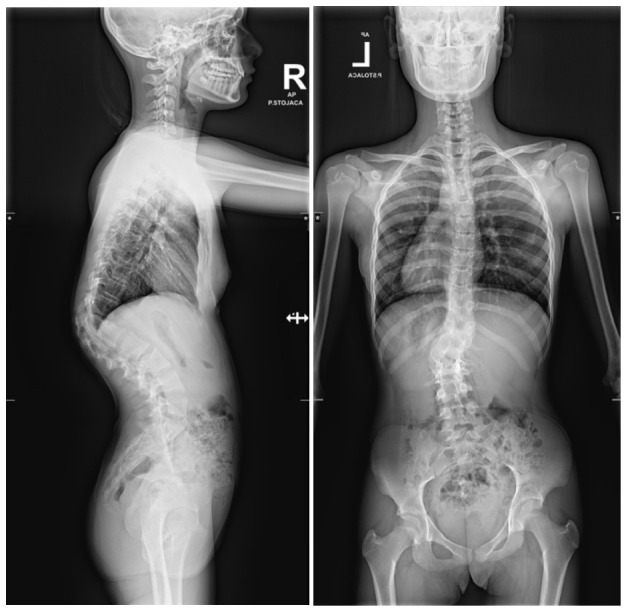
Standing posteroanterior and lateral full-spine radiography showed a congenital vertebral anomaly, consisting of a lower-thoracic (T11 level), leading to a progressive structural kyphoscoliotic deformity.

Standing radiographs illustrated a severe rigid thoracolumbar kyphosis measuring 92°, which was associated with congenital dysplasia of the T11 vertebral body and coronal imbalance (Figure 9). Flexibility assessments demonstrated minimal correction, with kyphosis improving only to 85° during traction radiographs. Magnetic resonance imaging (MRI) ruled out the presence of intraspinal anomalies but revealed significant narrowing of the spinal canal, dural sac compression, and stretching of the spinal cord at the apex of the deformity. Three-dimensional computed tomography (CT) confirmed the presence of severe anterior vertebral body deficiency at T11, elucidating the pronounced rigidity and the absence of anterior column support.

**Figure 10:**
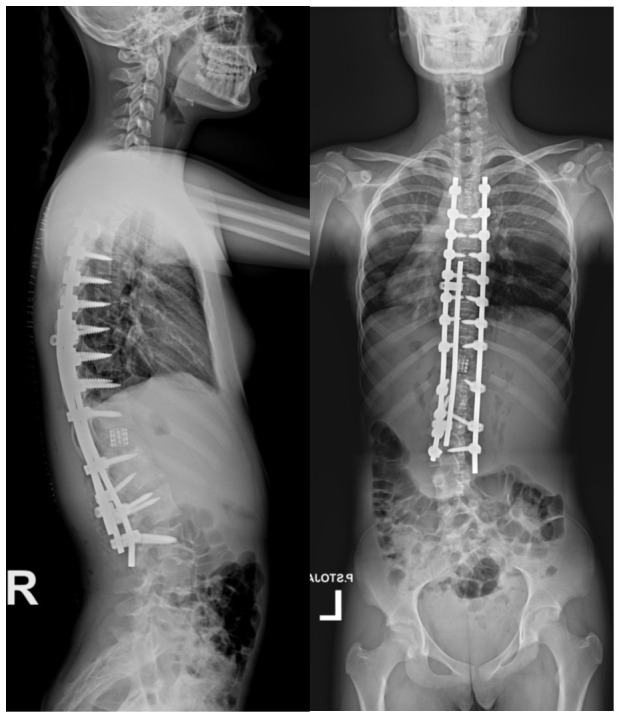
Standing posteroanterior and lateral full-spine radiographs obtained just after surgery (first standing X-rays).

**Figure 11:**
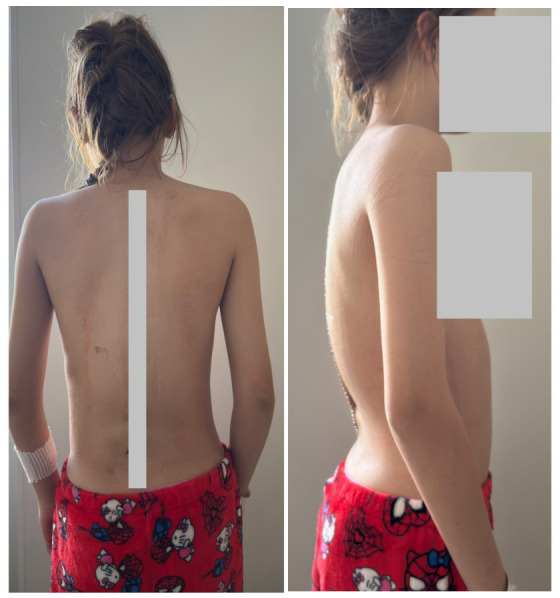
Standing clinical pictures obtained just after surgery.

In light of the progressive nature of the deformity, the severe rigidity, spinal canal compromise, and deficiency of the anterior column, posterior vertebral column resection (VCR) was deemed the most suitable corrective intervention [4,33,34,46,47,48,49,50]. Less extensive surgical options, such as Ponte osteotomies or pedicle subtraction osteotomy, were considered unlikely to yield sufficient correction. The surgical procedure encompassed the resection of the T11 vertebral column, anterior column reconstruction utilizing a titanium mesh cage, multilevel Ponte osteotomies, and posterior fusion extending from T4 to L3. Pedicle screw fixation was achieved using the Reline^®^ spinal system (Globus Medical, Inc., Audubon, PA, USA). The surgery was executed without complications while employing continuous neuromonitoring. The operative duration was recorded at 8.5 h, with an estimated blood loss of 1200 mL, and no transfusions were necessitated [51]. Postoperative radiographs confirmed a correction of kyphosis from 92° to 38° (representing a 59% correction), restoration of coronal balance, and satisfactory positioning of the cage without any indications of subsidence or migration. The postoperative trajectory was uneventful, and the patient was discharged on post-operative day 6 without any neurological deficits. The radiographic and clinical outcomes are illustrated in Figure 10 and Figure 11.

**Figure 12:**
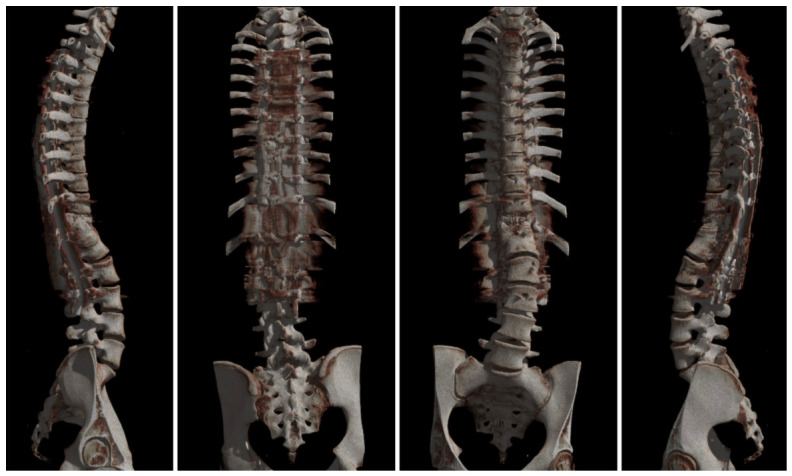
The imaging an CT obtained at a 2-year of follow-up showed posterior spinal fusion.

At three-month follow-up, the patient had returned to full mobility with excellent functional rehabilitation. She observed a significant improvement in both the cosmetic look of her back alongside her general quality of her life. Serial follow-up radiographic examinations showed a stable alignment of the spine with maintenance of correction without any sign of failure of hardware used. CT imaging during 2-year follow-up substantiated firm fusion, with excellent incorporation of the titanium cage mesh without any sign of pseudoarthrosis (as presented in Figure 12).

### 3.3. Comparison of Surgical Outcomes

All two subjects exhibited considerable radiographic amelioration and a noteworthy enhancement in health-related quality of life following surgical intervention (Table 1). The degree of kyphosis correction varied, with Case 1 achieving a 42% correction, while Cases 2 attained a 59% correction; additionally, the coronal deformity noted in Case 1 improved from 80° to 22°, representing a 72.5% correction (Table 2). Functional outcomes were significantly elevated, as evidenced by SRS-22R scores which escalated from a preoperative range of 3.0–3.8 to a final follow-up range of 4.6–4.8. In a similar vein, pain levels markedly diminished, with visual analog scale (VAS) scores transitioning from a preoperative range of 4–6/10 to a postoperative range of 0–1/10. Disability, quantified via the Oswestry Disability Index (ODI), showed improvement across all patients, decreasing from a preoperative range of 32–46 to a final follow-up range of 5–12. While VCR was correlated with extended operative durations and increased blood loss compared to the non-VCR approach, no instances of neurological complications, implant failures, or loss of correction were documented. Robust fusion and the preservation of correction were verified in all patients at the final follow-up.

Three-rod constructs were selectively used in cases requiring partial vertebral resection or extensive corrective maneuvers. The accessory rod was applied across the region of maximal correction to increase construct rigidity, improve load sharing, and reduce mechanical stress on the primary rods. In congenital spinal deformities, where abnormal vertebral anatomy and asymmetric loading increase the risk of implant failure, multi-rod constructs may enhance both immediate postoperative stability and long-term durability of fixation to further reinforce the osteotomy or resection site and reduce the risk of rod fracture and pseudarthrosis.

## 4. Discussion

Rigid or congenital deformities of the spinal column represent some of the most technically demanding challenges encountered within contemporary spinal surgical practice [1,2]. Severe deformities frequently necessitate aggressive corrective methodologies due to extreme structural rigidity, multi-planar deformities, and the presence of congenital lesions that disrupt the normal anatomical and biomechanical integrity of the spine [36,47,54,55,56]. Historically, substantial congenital spinal deformities have frequently been addressed through a combination of anterior release, anterior reconstruction, and posterior spinal fusion techniques. While these methodologies facilitate significant mobilization of inflexible curves and direct restoration of the anterior column, they are concomitant with heightened operative duration, increased blood loss, pulmonary complications, and greater surgical exposure. Progressions in posterior instrumentation, pedicle screw fixation, osteotomy techniques, and vertebral resection methodologies have permitted comparable corrective outcomes to be attained via posterior-only strategies in a considerable number of patients.

In the current instances, a posterior-centric approach was chosen to mitigate the morbidity linked with thoracotomy or retroperitoneal exposure while concurrently achieving adequate coronal and sagittal alignment. Although an integrated anterior–posterior surgical approach may present benefits in select cases of severe deformities, it is typically more invasive and correlated with elevated perioperative morbidity and extended hospitalization durations. Consequently, the decision-making process regarding the selection between combined and posterior-only strategies should be tailored to the individual characteristics of the deformity, the patient’s comorbid conditions, and the surgeon’s expertise. Vertebral Column Resection (VCR) and its posterior-only modification (PVCR) continue to be regarded as some of the most potent surgical interventions for the rectification of severe rigid spinal deformities. By excising all three columns of the spine at the apex of the deformity, VCR facilitates significant correction in both the coronal and sagittal planes, particularly in individuals with severe congenital anomalies, fixed kyphoscoliosis, post-traumatic deformities, or unsuccessful prior reconstructions [2,12,13,14]. Published studies have documented coronal correction rates ranging from 54% to 67% and sagittal correction rates from 60% to 91%, which typically surpass those attained through less extensive osteotomies. Nevertheless, these advantages are accompanied by heightened surgical complexity, extended operative duration, considerable blood loss, and an increased incidence of complications. Reported overall complication rates vary from 24% to 100%, while neurological complications are the most concerning adverse events, occurring in approximately 11–15% of contemporary series derived from high-volume medical centers [6,8,9,13,15]. Spinal correction is achieved through a controlled closure or opening of the lesion, with posterior instrumentation, consisting of rods, cages, and pedicle screws, utilized for the maintenance of stability. Technical advancements, including temporary rod stabilization, staged correction, anterior column reconstruction, and routine multimodal neuromonitoring, have enhanced the safety profile associated with VCR. The routine intraoperative application of motor-evoked potentials (MEPs) and somatosensory-evoked potentials (SSEPs) for neuromonitoring is employed to ensure comprehensive neurological protection. VCR is typically considered when less invasive treatment modalities have proven ineffective [5,12]. VCR remains a highly demanding yet remarkably efficacious surgical technique for the correction of inflexible, severe, or rigid spinal deformities, such as kyphosis, kyphoscoliosis, and neglected scoliosis [1,2,3,7,57,58]. Nevertheless, contemporary comparative investigations indicate that while the overall rates of complications may be analogous between vertebral column resection (VCR)-based and non-VCR-based methodologies [8,9,12,14,15,59,60,61,62], VCR is nonetheless correlated with a substantially elevated risk of neurological complications [30,31,63,64,65,66]. In addition to neurological complications, VCR is characterized by considerable intraoperative hemorrhage, with average blood loss ranging from 1500 milliliters to 3000 milliliters, accompanied by prolonged surgical durations that frequently exceed eight hours. Additional complications that may manifest as a consequence of VCR include instrumentation failure, cage migration, and the emergence of pseudoarthrosis [28,37,47,51,54,67]. Lewis, in his contributions from 2011, demonstrated robust correction rates, expedited postoperative mobility, and sustained sagittal alignment in cases of spinal deformities [68,69,70]. In spite of its inherent risks, VCR remains a fundamental and indispensable technique for addressing fixed, severe spinal deformities. With meticulous surgical planning, stringent adherence to advanced refinements, and the integration of neuromonitoring, contemporary VCR for highly pathological and complex spinal pathology patients yields transformative outcomes. The recognition of the significant risks associated with VCR has precipitated the development and refinement of alternative approaches that potentially achieve effective correction while mitigating operative morbidity. These alternatives have generally been categorized into various classifications: preoperative traction methods, staged surgical interventions, modified osteotomy techniques, and temporary distraction [1,2,3,4,41].

Halo-gravity traction (HGT) constitutes one of the most established adjunctive modalities in the treatment of severe rigid spinal deformities [43,44,45]. Initially devised to facilitate gradual correction of deformities through sustained axial traction, HGT persists as a significant preoperative strategy in contemporary deformity surgery. The methodology employs a halo ring that is affixed to the cranial structure and linked to incrementally increasing weights, generally applied for a duration of 3 to 12 weeks. Throughout this interval, traction enhances spinal flexibility, partially ameliorates the deformity, promotes spinal cord adaptation, and augments patient tolerance to definitive surgical intervention [4,42,71]. The principal indications for HGT encompass severe curves surpassing 100°, compromised pulmonary function (notably FVC < 40% predicted), augmented neurological risk, and deformities that might otherwise necessitate highly invasive corrective measures such as vertebral column resection (VCR) [10,72,73,74]. By progressively elongating the spinal cord, nerve roots, and adjacent soft tissues, HGT may mitigate the risk of neurological injury during subsequent corrective procedures [72,75,76]. Numerous investigations have substantiated that HGT enhances curve flexibility, pulmonary function, and overall surgical preparedness [43,74,75,77]. A recent meta-analysis disclosed considerable enhancements in deformity magnitude and respiratory function, while additional studies have reported improvements in thoracic height, nutritional status, and body weight during the traction phase [43,74,75,77]. These advantages may facilitate definitive correction, minimize blood loss, and lessen the necessity for extensive osteotomies [43,74]. Complications associated with HGT are generally minor and comprise pin loosening and superficial infections at the pin sites. Although a meta-analysis indicated an overall complication rate of approximately 22%, the majority of adverse events were transient and managed conservatively, with permanent neurological complications being infrequent [10,72]. While traction protocols differ among medical institutions, the majority of deformity correction occurs within the initial 4 to 6 weeks of treatment [77,78]. Recent studies have also indicated that outpatient HGT may yield comparable rates of correction and complications to inpatient protocols, concurrently reducing healthcare costs in suitably selected patients [45,79].

Temporary Internal Distraction Rods (TIDRs) constitute a relatively novel approach in the management of severe rigid spinal deformities [80,81]. This technique employs temporary internal rods that traverse the apex of the deformity, facilitating gradual correction over a span of days to weeks through meticulously controlled distraction. TIDRs build upon the foundational principles of halo-gravity traction while simultaneously offering the benefits of internal fixation, thereby obviating the necessity for external traction apparatus [45,48,49,82]. TIDRs are particularly advantageous for patients exhibiting severe deformities (Cobb angle > 90°), wherein single-stage corrective measures present an augmented risk of neurological compromise and mechanical complications [48]. They provide a regulated alternative that lies between preoperative traction and more invasive interventions, such as three-column osteotomies or vertebral column resection (VCR) [35,41,45,82,83]. The implementation of this technique is generally executed in two distinct stages. The initial stage encompasses posterior instrumentation and the installation of temporary distraction rods across the apex of the deformity, succeeded by a period of gradual correction. In the subsequent stage, the temporary rods are extracted, and definitive instrumentation is instituted, with additional osteotomies performed as clinically indicated [41,80,81]. Such a staged methodology facilitates repeated neuromonitoring and progressive acclimatization of the spinal cord and adjacent soft tissues, potentially mitigating neurological risks [34]. Numerous studies have documented favorable outcomes associated with TIDRs. Buchowski et al. evidenced correction rates surpassing 60% without the occurrence of permanent neurological deficits [48], while Skaggs et al. validated the safety of gradual distraction despite sporadic reversible neuromonitoring alerts [49]. Grabala et al. reported considerable deformity correction alongside enhancements in health-related quality of life through the application of staged distraction techniques [45]. In a similar vein, Hu et al. illustrated the safe correction of curves exceeding 130°, concurrently diminishing the necessity for high-risk three-column osteotomies and reducing operative time and intraoperative blood loss [34]. Notwithstanding these advantages, the utilization of TIDRs necessitates staged surgical interventions, which consequently escalate resource utilization and prolong the overall treatment timeline. Factors such as mechanical failure, rod loosening, infection, suboptimal bone quality, and pre-existing neurological deficits may constrain their applicability in select patient populations [4,34,45,48,49,82].

Magnetically Controlled Growing Rods (MCGRs), initially conceived for the treatment of early-onset scoliosis, have progressively been repurposed as a temporary internal distraction technique for individuals afflicted by severe rigid spinal deformities [84]. The primary benefit of this technology lies in its capacity to facilitate gradual, non-invasive distraction via an internal magnetic mechanism that is externally regulated, thus enabling progressive correction of deformities and enhancement of spinal flexibility without necessitating repeated surgical interventions or the use of external traction apparatus [41,83]. In contrast to halo-gravity traction (HGT), MCGR provides a comprehensive internal distraction approach, thereby circumventing the need for cranial pins, external fixation, and extended periods of hospitalization [43,74,75]. Patients maintain ambulation throughout the distraction phase, which may contribute to an enhanced quality of life and a reduction in complications associated with traction [1,2,45]. MCGR has been particularly beneficial for patients exhibiting severe spinal curves (>80–90°), confirmed rigidity on bending films, contraindications for HGT, or intricate revision deformities necessitating staged corrective procedures [41,45,62,83]. Clinical evidence indicates that distraction utilizing MCGR diminishes curve rigidity, enhances trunk equilibrium, and may permit less extensive definitive surgical interventions, in alignment with the principle of gradual preoperative deformity conditioning [41,45,62,83]. Nonetheless, the application of this technique necessitates two surgical interventions for both insertion and removal, and it is accompanied by potential complications, including actuator malfunction, rod displacement, prominence, MRI artifacts, and elevated initial implant expenses [41,45,62,83]. While HGT continues to be the preferred modality when maximal three-dimensional correction and intensive inpatient rehabilitation are essential, MCGR represents a significant alternative for carefully selected patients, facilitating gradual internal correction with commendable tolerability and diminished morbidity related to traction [41,45,62,83].

Asymmetric Pedicle Subtraction Osteotomy (APSO) represents an advancement of the conventional Pedicle Subtraction Osteotomy (PSO), specifically engineered to rectify severe multiplanar spinal deformities via a singular osteotomy site [5,59,60,85,86]. In contrast to traditional PSO, which predominantly rectifies sagittal imbalance, APSO employs asymmetric wedge resection, thereby facilitating concurrent correction in both the sagittal and coronal planes [5,59,60,85,86]. Consequently, APSO has emerged as a less invasive alternative to Vertebral Column Resection (VCR) for select patients presenting with intricate spinal deformities [9,10,13,15,16]. The primary indications for APSO encompass fixed sagittal imbalance, coronal decompensation, adult degenerative scoliosis, residual deformity subsequent to prior fusion, and specific instances of rigid scoliosis necessitating simultaneous correction in two planes [7,30,59,61,87,88]. By facilitating significant correction through a singular osteotomy level, APSO has the potential to diminish operative morbidity while maintaining enhanced spinal stability in comparison to more extensive surgical interventions [27,28,54,61,88]. Documented correction rates are reported to range from 25° to 35° per level in the sagittal plane, while approximately 10° to 20° is observed within the coronal plane [54,55,56]. The incidence of neurological complications remains relatively low, with transient deficits occurring in 2–5% of patients and permanent deficits in less than 1%, which is substantially lower than those documented for VCR [32,40]. When juxtaposed with PSO, APSO yields comparable sagittal correction accompanied by additional correction in the coronal plane, whereas in comparison with VCR, it is associated with diminished blood loss, reduced operative duration, and a lower overall complication profile [32,40,47,89]. Although data regarding long-term outcomes remain sparse, APSO appears to serve as an effective intermediate option bridging conventional PSO and VCR, providing considerable multiplanar correction with reduced surgical morbidity in appropriately selected patients [32,40,47,55,56,68,69,70,89].

Multi-rod constructs have assumed an increasingly pivotal role in the management of severe congenital spinal deformities. By extending across osteotomy or vertebral resection sites with additional rods, these constructs enhance load distribution, mitigate rod strain, and augment construct longevity. This consideration is particularly pertinent in congenital deformities, where atypical vertebral morphology, substantial corrective forces, and extensive fusion constructs heighten the likelihood of rod fracture and pseudarthrosis. Biomechanical investigations have elucidated that multi-rod constructs enhance stability and diminish stresses at osteotomy and resection locations; however, the principle of optimized load sharing, as opposed to the maximization of construct rigidity, emerges as the principal biomechanical tenet [90]. Clinical investigations have similarly revealed diminished rates of rod fracture and enhanced preservation of postoperative alignment when employing three- or four-rod constructs in comparison to conventional two-rod configurations [91,92]. Recently, reinforced multi-rod constructs featuring up to six rods have exhibited encouraging preliminary outcomes in complex deformity surgery, with no rod fractures reported during the follow-up period [93]. Collectively, these observations advocate for the implementation of accessory rods in instances of severe deformities necessitating substantial correction, osteotomy, or vertebral resection, thereby promoting construct resilience and the prolonged maintenance of correction [91,92,93].

The optimal density of implants utilized in spinal deformity surgery continues to be a subject of significant debate within the medical community. While high-density pedicle screw constructs may enhance immediate corrective efficacy and structural rigidity, they concomitantly incur increased expenses for implants, extended duration of surgical procedures, and elevated levels of hemorrhage. Biomechanical investigations have elucidated that augmenting screw density does not inherently result in enhanced correction of coronal, sagittal, or axial deformities; instead, the contouring of rods and the strategic allocation of implants may exert a more profound impact on achieving optimal alignment [94]. Clinical data also raises doubts regarding the habitual application of maximal implant density. Comparative analyses, multicenter research cohorts, and recent systematic reviews have consistently indicated that radiographic correction, complication rates, and patient-reported outcomes are comparable across low-, moderate-, and high-density constructs [95,96,97,98]. Concurrently, instrumentation characterized by lower density has been correlated with diminished surgical duration, reduced hemorrhage, and significantly lower costs associated with implants [95,96,97,98]. Numerous studies have indicated that implant densities surpassing approximately 60–70% do not yield clinically significant enhancements in deformity correction, notwithstanding the increased financial burden [97,98]. As a result, contemporary practices in deformity surgery increasingly advocate for an optimized distribution of implants rather than the indiscriminate maximization of screw density. In cases of severe congenital deformities, the durability of constructs relies not solely on implant density but also on the implementation of suitable load-sharing strategies and the incorporation of auxiliary rods at osteotomy or vertebral resection sites [90,91,92,93]. Evaluating cost differentials among various surgical methodologies remains complex, as implant pricing and healthcare systems exhibit substantial variability across different institutions and nations. Nonetheless, a comprehensive assessment of the overall economic implications should consider not only the costs of implants but also factors such as surgical duration, blood loss, length of hospital stays, management of complications, necessitated revision surgeries, and the long-term durability of constructs. Table 3 provides a practical comparison of the principal surgical strategies currently used for severe rigid spinal deformities, summarizing their expected correction potential, complication profile, cost considerations, and hospitalization requirements developed based on the literature [1,2,3,4,5,6,7,8,9,10,11,12,13,14,15,16,17,18,19,20,21,22,23,24,25,26,27,28,29,30,31,32,33,34,35,36,37,38,39,40,41,42,43,44,45,46,47,48,49,50,51,54,55,56,57,58,59,60,61,62,63,64,65,66,67,68,69,70,71,72,73,74,75,76,77,78,79,80,81,82,83,84,85,86,87,88,89,99,100,101,102,103,104,105,106,107,108,109]. Although these estimates are derived from the available literature and should be interpreted cautiously, they may assist surgeons in selecting the most appropriate treatment strategy based on deformity characteristics, patient-specific factors, and institutional resources.

### Limitations

This study has several limitations. First, it presents only three illustrative cases and therefore does not allow for definitive conclusions regarding the superiority of one surgical strategy over another. Second, the literature review was narrative rather than systematic and is therefore susceptible to selection bias. Third, direct comparisons between different surgical techniques are limited by the heterogeneity of patient populations, deformity characteristics, surgical indications, and outcome measures reported in the available literature. Finally, long-term comparative data evaluating functional outcomes, fusion rates, patient satisfaction, and cost-effectiveness across different treatment strategies remain limited.

Despite these limitations, the cases presented in this study illustrate that the management of congenital rigid spinal deformities should not be viewed as a simple choice between VCR and non-VCR techniques. Rather, successful treatment depends on selecting the least invasive strategy capable of achieving acceptable deformity correction while maintaining neurological safety and long-term construct stability. The increasing availability of staged correction techniques, advanced osteotomies, preoperative conditioning strategies, and multi-rod constructs has expanded the surgical armamentarium and reduced the need for routine vertebral column resection in selected patients. The existence of various surgical methodologies for the correction of rigid spinal deformities necessitates a tailored and methodical approach to decision-making [5,6,7,8,32,36,40,46,82]. The formulation of a treatment plan should commence with a thorough evaluation of the deformity’s flexibility, the neurological condition of the patient, and the specific risk factors pertinent to the individual, employing standing and dynamic radiographic imaging, traction films, and clinical assessments [4,32,40,41,46,68,69,82,83]. In individuals presenting with considerable rigid deformities, preoperative preparation through the application of HGT, TIDRs, or MCGRs may enhance flexibility and facilitate subsequent corrective interventions [4,32,40,41,46,68,69,82,83]. The surgical rectification should thereafter be conducted in a systematic manner, initiating with less invasive procedures such as Ponte or Smith-Petersen osteotomies. Should these techniques fail to yield satisfactory correction, more invasive options such as PSO or APSO may be contemplated. VCR should typically be reserved for the most severe deformities, especially when less invasive interventions are deemed insufficient for achieving adequate correction or when complete vertebral destabilization is warranted [5,6,7,8,32,36,40,46,82]. This incremental strategy aspires to optimize the correction of the deformity while concurrently minimizing the associated surgical morbidity [37,39,41,83,84,109,110,111]. Although the quantity of direct comparative studies remains limited, existing evidence indicates that the utilization of preoperative traction and staged correction methodologies can diminish surgical complexity and, in select patient populations, obviate the necessity for VCR while preserving acceptable clinical and radiographic results [33,34,48,49,72,73,74,75,78].

## 5. Conclusions

Vertebral Column Resection (VCR) remains an important surgical option for the treatment of severe rigid spinal deformities; however, it should not be considered the routine treatment strategy for all patients. Contemporary alternatives, including preoperative traction, staged distraction techniques, and advanced osteotomies such as APSO or PSO, may achieve satisfactory deformity correction with lower morbidity in appropriately selected cases. Successful management requires individualized treatment planning based on deformity severity, spinal flexibility, neurological risk, patient comorbidities, and functional goals. In high-risk patients, the primary objectives should focus on improving function, quality of life, and preventing deformity progression rather than achieving maximal radiographic correction. A stepwise treatment strategy, supported by multidisciplinary evaluation and modern intraoperative neuromonitoring, may optimize outcomes while minimizing surgical risk. VCR should therefore be reserved for the most severe and complex deformities in which less invasive techniques are unlikely to provide adequate correction.

## 6. Clinical Recommendation

Management of severe rigid congenital spinal deformities should be individualized based on deformity morphology, flexibility, neurological status, patient comorbidities, and treatment goals. Preoperative conditioning strategies, including halo-gravity traction (HGT), temporary internal distraction rods (TIDRs), or magnetically controlled growing rods (MCGRs), should be considered in selected patients with severe rigid deformities to improve flexibility and reduce the need for extensive corrective procedures. Less invasive corrective techniques, such as Ponte osteotomies, PSO, or APSO, should be considered before vertebral column resection whenever satisfactory correction can be achieved safely. Vertebral column resection remains an important option for severe rigid deformities with marked vertebral dysplasia, anterior column deficiency, fixed angular kyphosis, or when less invasive techniques are unlikely to provide adequate correction. Multi-rod constructs should be considered in cases requiring major correction, osteotomy, or vertebral resection to improve construct durability and reduce the risk of mechanical failure. Surgical success should be evaluated not only by radiographic correction but also by neurological safety, functional improvement, pain reduction, and patient-reported outcomes.

## Figures and Tables

**Table 1:** HRQoL of treated patients.

Questionnaire Outcomes	Patient 1 (Case 1)	Patient 2 (Case 2)
SRS-22R total score, preoperatively	3.6	3.8
SRS-22R total score, at the final follow-up	4.8	4.8
VAS score, preoperatively	4/10	4/10
VAS score, at the final follow-up	0/10	0/10
ODI score, preoperatively	38	32
ODI score, at the final follow-up	6	5

The SRS-22r questionnaire was used, with visual analogue scale (VAS) score and Oswestry Disability Index (ODI) parameters collected preoperatively and at the final follow-up [52,53].

**Table 2:** Pre- and postoperative parameters for all treated patients.

Kyphosis Correction	72° → 42° (42%)	92° → 38° (59%)
Coronal correction	80° → 22° (72.5%)	N/A
Operative time (h)	6.5	8.5
Blood loss (mL)	800	1200
Fusion length	T4–L4	T4–L3

N/A: Not Applicable.

**Table 3:** Expected correction, neurological risk, cost, and length of stay represent relative estimates derived from the available literature and may vary according to deformity type, patient characteristics, and institutional practice [1,2,3,4,5,6,7,8,9,10,11,12,13,14,15,16,17,18,19,20,21,22,23,24,25,26,27,28,29,30,31,32,33,34,35,36,37,38,39,40,41,42,43,44,45,46,47,48,49,50,51,54,55,56,57,58,59,60,61,62,63,64,65,66,67,68,69,70,71,72,73,74,75,76,77,78,79,80,81,82,83,84,85,86,87,88,89,99,100,101,102,103,104,105,106,107,108,109].

Technique	Main Indications	Expected Correction	Neurological Risk	Advantages	Limitations	Relative Cost	Length of Stay
PSF Alone	Flexible or moderately rigid deformities	Low to moderate	Low	Lowest morbidity, shorter surgery	Limited correction in severe rigid curves	Low	Short
AR+ PSF	Severe rigid deformities requiring anterior mobilization	Moderate to high	Moderate	Improved flexibility, anterior column support	Thoracic/abdominal morbidity, increased blood loss	High	Moderate–Long
HGT	Severe rigid deformities (>90–100°), poor pulmonary reserve	Indirect preoperative correction	Low	Improves flexibility, pulmonary function, nutritional status	Pin-related complications, prolonged treatment	Moderate	Long
TIDRs	Very severe rigid deformities requiring staged correction	High	Low–Moderate	Gradual correction, reduced neurological stress	Two-stage procedure, increased resource utilization	High	Long
MCGRs	Severe rigid deformities requiring gradual internal distraction	Moderate to high	Low–Moderate	Internal distraction, outpatient lengthening	High implant cost, limited availability	Very High	Moderate
Ponte/SPO	Moderately rigid deformities	Moderate	Low	Less invasive, preserves stability	Limited corrective power in severe deformities	Moderate	Short–Moderate
PSO	Fixed sagittal imbalance	High sagittal correction	Moderate	Powerful correction through single osteotomy	Blood loss, technical complexity	High	Moderate
APSO	Multiplanar rigid deformities	High coronal and sagittal correction	Low–Moderate	Lower morbidity than VCR, multiplanar correction	Technically demanding	High	Moderate
VCR	Most severe, rigid, multiplanar deformities	Very High	Highest	Greatest corrective potential	Highest blood loss, neurological risk, operative complexity	Very High	Long

## Data Availability

Data are contained within the article.
